# Genomic risk factors for central nervous system relapse in patients with diffuse large B-cell lymphoma

**DOI:** 10.1007/s44313-025-00087-1

**Published:** 2025-07-01

**Authors:** Shiyu Jiang, Qunling Zhang, Jia Jin, Wenhao Zhang

**Affiliations:** 1 Department of Medical Oncology, Fudan University Shanghai Cancer Center, 270 Dongan Rd, Shanghai, 200032 China; 2 Research Center for Lymphoma, Fudan University, 270 Dongan Rd, Shanghai, 200032 China

**Keywords:** Diffuse large B-cell lymphoma, Central nervous system, Relapse, Risk, Mutation

## Abstract

**Purpose:**

Central nervous system (CNS) relapse is associated with poor survival, and remains an unmet challenge in patients with diffuse large B-cell lymphoma (DLBCL). Identifying patients at high risk of CNS relapse and offering prophylactic treatment could improve patient prognosis.

**Methods:**

Here, we studied 234 patients with DLBCL using open patient-level clinical and sequencing data to explore risk factors for CNS relapse. Patients were divided into Cohort A (CNS involvement at baseline), Cohort B (CNS recurrence), and Cohort C (patients without secondary CNS involvement and with a follow-up interval > 3 years). We investigated the risk factors for CNS relapse in Cohorts B + C.

**Results:**

Genetic alterations with statistical significance, determined by univariate analysis, and an incidence rate ≥ 5%, together with clinical factors, correlated with CNS relapse risk in a multivariate analysis. Multivariate logistic regression analysis revealed that concomitant *MYD88 *L265P and *CDKN2A* loss (*p* = 0.012), *TET2* mutation (*p* = 0.037), *ARID1A* mutation (*p* = 0.010), and *INO80* (*p* = 0.002) were independently correlated with a high risk of CNS relapse after adjusting for the IPI risk groups, B symptom and cell of origin (COO). The classifier that integrated genomic risk factors was superior in predicting CNS relapse (area under the receiver operating characteristic curve [AUROC]: 0.91) compared with the IPI (AUROC: 0.77, *p* < 0.001) or IPI in combination with COO classifiers (AUROC: 0.81, *p* = 0.013).

**Conclusion:**

This study identified several genomic alterations as risk factors for CNS relapse.

**Supplementary Information:**

The online version contains supplementary material available at 10.1007/s44313-025-00087-1.

## Introduction

Diffuse large B-cell lymphoma (DLBCL) is a heterogeneous entity, with over half of patients cured and the remaining patients experiencing relapse or refraction [8, 16]. Overall, approximately 5% of patients with DLBCL will develop central nervous system (CNS) events (relapse or progression), resulting in unfavorable responses and adverse outcomes [13, 23, 26, 32]. According to previous reports, patients with DLBCL with secondary CNS relapse have an overall survival (OS) of 3.5–7.0 months following the diagnosis of CNS occurrence, which remains a great unmet medical need [20, 22]. Therefore, in addition to early diagnosis and CNS-directed interventions, it is critical to identify patients at a high risk of CNS relapse and apply prophylactic strategies.

Effort has been devoted to the identification of patient risk for CNS recurrence, and a growing body of evidence has indicated several clinical parameters as risk factors, such as increased serum lactate dehydrogenase (LDH) levels, involvement of ≥ 2 extranodal sites, and the International Prognostic Index (IPI) score [1–3, 5, 6, 11]. With the addition of rituximab to the commonly used chemotherapy treatment with cyclophosphamide, doxorubicin, vincristine, and prednisone (CHOP), a reduced 2-year incidence of CNS disease from 6.9% to 4.1% has been observed, which could probably be due to a superior control of the systemic disease [7, 17]. Other risk factors for CNS recurrence include specific site involvement (testis, breast, and kidney) [4, 9, 10]. Nevertheless, despite investigations into risk stratification and the application of prophylactic interventions, CNS relapse remains a clinical challenge in the post-rituximab era.

Originally constructed based on a large dataset of clinical trial participants and validated in independent DLBCL cohorts, the CNS-IPI has been proposed to assess the risk of CNS recurrence in DLBCL, which includes five IPI factors plus kidney/adrenal gland involvement. This model stratifies the patients into three risk groups. The low-risk group (0–1 factors) has an estimated 2-year incidence of CNS relapse of 0.6%, while the proportions are higher in the intermediate (2–3 factors) and high-risk (4–6 factors) groups, with 2-year risks of 3.4% and 10.2%, respectively [22]. Since its publication, the CNS-IPI has produced remarkably consistent risk estimates across different studies and has emerged as the only index recommended by the National Comprehensive Cancer Network (NCCN) guidelines to assess CNS relapse risk. However, the percentage of high-risk patients with 2-year CNS involvement in DLBCL ranges between 10.2% and 12.9%, according to the CNS-IPI, which confers modest positive and high negative predictive values to this group of patients [22, 27].

Owing to an understanding of the biology of DLBCL, the cell of origin (COO), *MYC* arrangement with either additional *BCL-2* or *BCL-6* gene rearrangements, and dual expression have been identified as adverse prognostic indicators for de novo DLBCL as well as for secondary CNS involvement in DLBCL [15, 21]. The Spanish Lymphoma Group (GELTAMO) recommends CNS prophylaxis for patients based on the presence of CNS disease according to the IPI and evolving risk factors, such as increased serum LDH; > 1 extranodal site involvement; extranodal involvement of the testis or breast; extranodal involvement of the kidney, adrenal gland, or epidural space; high-risk CNS-IPI scores; and *MYC* rearrangements associated with *BCL2* or *BCL6* rearrangements [24].

The impact of genomic alterations and genotyping on the prognosis of patients with DLBCL have been extensively explored. Importantly, differences in genomic alterations in primary central nervous system lymphoma (PCNSL) and DLBCL, not otherwise specified (NOS) have been addressed [31]. Nevertheless, the differences between patients experiencing CNS relapse and those without relapse have rarely been explored. Recently, Klanova et al. indicated that *CDKN2A* and *MYD88* mutations occur more frequently in populations with CNS relapse [29]. However, data on the association between genomic features and the risk of CNS recurrence in DLBCL are limited. We conducted this study to identify biomarkers associated with CNS relapse risk in patients with DLBCL, willing to optimize CNS risk stratification.

## Material and methods

### Data and study design

Open patient-level clinical data were obtained from a study including 1001 patients with DLBCL that were treated with a rituximab-containing standard regimen in the first-line setting. Such study included an integrative analysis of whole exome sequencing and transcriptome sequencing [25]. The clinical information and genetic alteration data for the 1001 DLBCL samples are listed in the supplementary material of such previous study (10.1016/j.cell.2017.09.027) [25]. The study design has been previously reported [25].

**Fig. 1:**
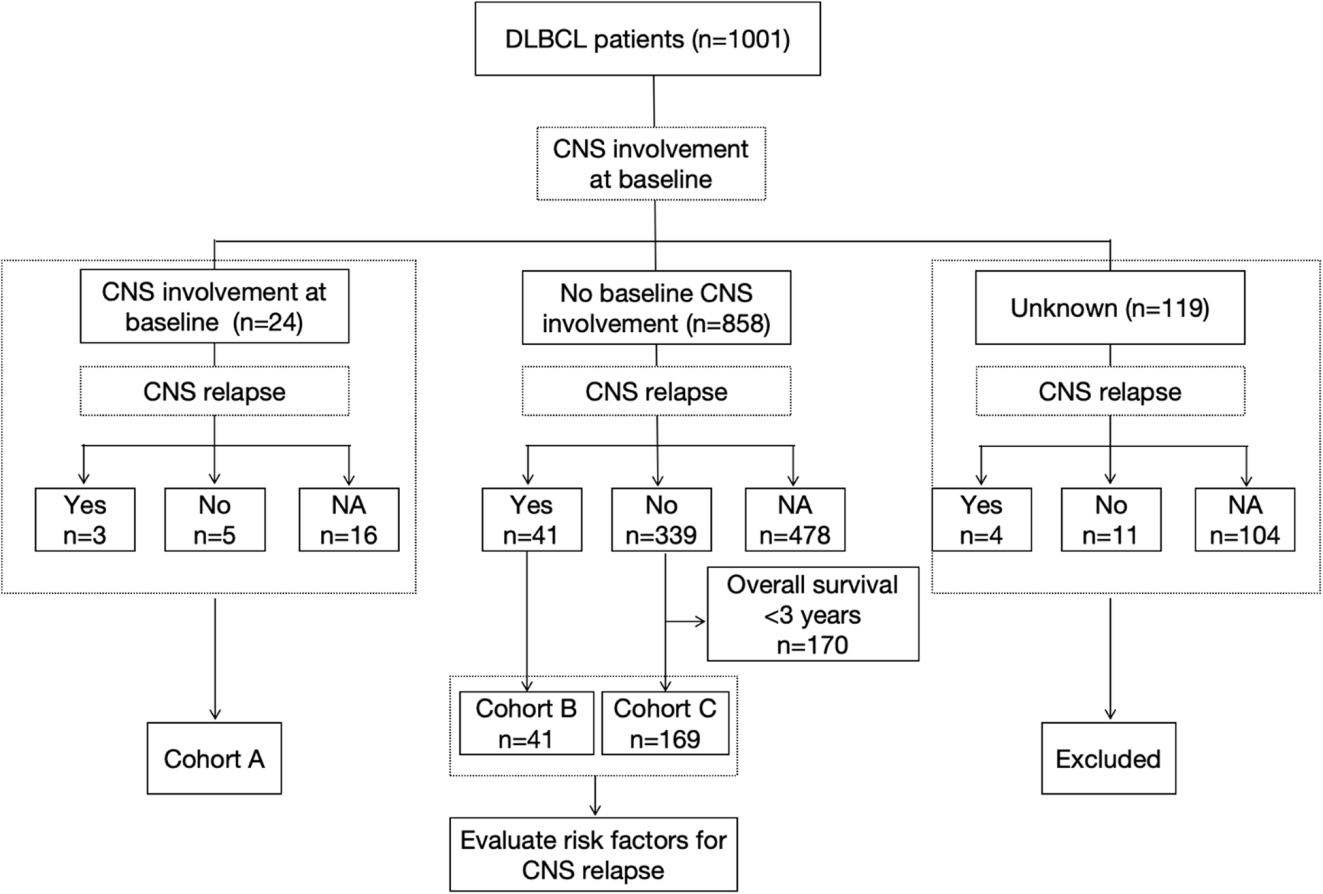
Study flowchart

The following clinical information, included in the supplementary file of the study, was extracted: age; sex; IPI and the binary label for each of the 5 components that comprise it; B symptoms at diagnosis; treatment response; testicular involvement; CNS involvement; CNS relapse; *MYC*, *BCL2*, and *BCL6* expression based on log2 transformation of RNA-seq fragments per kilobase (FPKM) value and high/low expression flags; *MYC*, *BCL2*, and *BCL6* translocation, assayed by fluorescence in situ hybridization (FISH); and COO classification based on gene expression profiling (GEP), NanoString classification, and Hans algorithm. All the clinical information and sequencing results presented in the supplementary files were reanalyzed in the current study. Overall, 24 patients had CNS involvement at baseline (Cohort A). Among 858 patients without primary CNS involvement, 380 had information on CNS events. To investigate the risk factors for CNS relapse in these 380 patients, we excluded those who were followed up for less than 3 years and without evidence of CNS involvement. The endpoint was 3-year CNS relapse. The 41 patients with CNS recurrence were categorized as Cohort B. Among the remaining 339 patients, 169 with a follow-up interval of more than 3 years were included in Cohort C. A flowchart of the study is shown in Fig. 1.

### Statistical analysis

We used Pearson’s χ^2^ test or Fisher’s exact test to compare the clinical/pathologic features between the groups. Differences in mutation prevalence were determined either by Fisher’s exact test or Pearson’s χ^2^ test. A logistic regression analysis was used to analyze risk factors for CNS relapse. The Chi-square test was used to examine the association between *MYD88* L265P and *CDKN2A* *loss*. All statistical analyses were performed using SPSS (version 22.0; IBM, Armonk, NY, USA) and R (version 3.0.2; http://www.R-project.org). Statistical significance was defined as a two-sided *p*-value < 0.05.

### Ethical statement

Not applicable.

## Results

### CNS events and patient clinical features

Overall, 234 patients were included in the current study, with 24, 41, and 169 patients in Cohorts A, B, and C, respectively (Fig. 1 ). More than half of the patients (124/234) were 60 years old and older. Most patients (69.2%, 162/234) had a good physical status, with an Eastern Cooperative Oncology Group (ECOG) performance status (PS) score of 0–1. At diagnosis, 53.4% (125/234) of the patients had elevated LDH levels and 21.8% (51/234) had two or more extranodal involvements. Besides, 80 (34.2%), 90 (38.5%), and 40 (17.1%) patients were classified in the IPI low-, medium-, and high-risk groups, respectively. Following the initial rituximab-containing regimen, most patients with available information (92.3%, 216/234) responded to the initial treatment, whereas 10 patients (4.3%) failed.

Patients with CNS relapse (Cohort B) had a higher IPI-based risk (*p* < 0.001). More specifically, individuals in Cohort B had worse PS (*p* < 0.001), higher advanced stages of the disease (*p* < 0.001), more frequent abnormal LDH levels (*p* = 0.001), and multiple extranodal involvement (*p* < 0.001) compared with those in Cohort C (Table 1). Although the COO distribution assessed by GEP between the two cohorts was not significantly different (*p* = 0.142), Cohort B was predominantly activated B cell-like (ABC, 46.3%; germinal center B cell-like [GCB], 24.4%), while Cohort C was equally distributed (ABC, 34.9%; GCB, 34.9%). Clinical features stratified by CNS involvement are shown in Table 1.

**Table 1:** Clinical features of patients with DLBCL, stratified in accordance with CNS involvement

Characteristics	Patient stratification by CNS involvement		
	Cohort A (*n* = 24) No. (%)	Cohort B (*n* = 41) No. (%)	Cohort C (*n* = 169) No. (%)
IPI groups			
Low (0–1)	2 (8.3)	1 (2.4)	77 (45.6)
Medium (2–3)	10 (41.7)	18 (43.9)	62 (36.7)
High (4–5)	4 (16.7)	14 (34.1)	22 (13.0)
Missing	8 (33.3)	8 (19.5)	8 (4.7)
ECOG PS			
0–1	12 (50.0)	16 (39.0)	134 (79.3)
≥ 2	7 (29.2)	18 (43.9)	34 (20.1)
Missing	5 (20.8)	7 (17.1)	1 (0.6)
Ann Arbor stage			
I/II	3 (12.5)	6 (14.6)	83 (49.1)
III/IV	18 (75.0)	34 (82.9)	85 (50.3)
Missing	3 (12.5)	1 (2.4)	1 (0.6)
Elevated LDH			
Yes	11 (45.8)	29 (70.7)	85 (50.3)
No	10 (41.7)	6 (14.6)	79 (46.7)
Missing	3 (12.5)	6 (14.6)	5 (3)
Extranodal sites			
0–1	14 (58.3)	24 (58.5)	143 (84.6)
≥ 2	10 (41.7)	16 (39.0)	25 (14.8)
Missing	0	1 (2.4)	1 (0.6)
Age > 60			
Yes	14 (58.3)	27 (65.9)	83 (49.1)
No	7 (29.2)	14 (34.1)	85 (50.3)
Missing	3 (12.5)	0	1 (0.6)
Response to initial therapy			
Complete response	13 (54.2)	29 (70.7)	150 (88.8)
Partial response	0	9 (22.0)	15 (8.9)
No response	4 (16.7)	3 (7.3)	3 (1.8)
Missing	7 (29.2)	0	1 (0.6)
Testicular involvement			
Female	8 (33.3)	16 (39.0)	74 (43.8)
No	15 (62.5)	17 (41.5)	49 (29.0)
Yes	1 (4.2)	6 (14.6)	10 (5.9)
Missing	0	2 (4.9)	36 (21.3)
COO (RNA-seq)			
ABC	11 (45.8)	19 (46.3)	59 (34.9)
GCB	5 (20.8)	10 (24.4)	59 (34.9)
Unclassified	2 (8.3)	2 (4.9)	23 (13.6)
Missing	6 (25.0)	10 (24.4)	28 (16.6)

ABC, activated B-cell-like; COO, cell of origin; ECOG PS, Eastern Cooperative Oncology Group performance status; GCB, germinal center B-cell-like; IPI, international prognostic index; LDH, lactate dehydrogenase; n, number of patients. Either Pearson’s χ2 test or Fisher’s exact test were used

### CNS events and mutational profile

The most common mutations in the three cohorts were analyzed. In Cohort A, *MYD88* (41.7%) was the most frequently mutated gene, followed by *MLL2* (20.8%) and *PIM1* (20.8%). Similarly, in Cohort B, *MLL2* (26.8%) and *MYD88* (26.8%) were the most frequently mutated genes. In Cohort C, mutations in *MLL2* (24.3%), *PIM1* (17.8%) and *MYD88* (15.4%) were the most common. We further analyzed the differences in mutation frequency by COO subtype (ABC vs. GCB) in Cohort B (Table 2), which showed that patients with ABC favored *MYD88* mutation (*p* = 0.027) and patients with GCB favored *TET2* mutation (*p* = 0.033). No difference in the co-overexpression of *MYC* and *BCL2* was observed between Cohorts B and C (*p* = 0.609). The mutational profiles were presented in Supplementary Files 1 and 2.

**Table 2:** Differences in mutation frequencies by COO subtype (ABC vs. GCB) in Cohort B

Gene	Stratified by COO		*p*-value
	ABC (*n* = 19) Occurrence (%)	GCB (*n* = 10) Occurrence (%)	
*MYD88*	8 (42.1)	0	0.027
*MYD88* L265P	6 (31.6)	0	0.068
*PIM1*	6 (31.6)	0	0.068
*TET2*	0	3 (30.0)	0.033

COO, cell of origin; ABC, activated B-cell-like; GCB, germinal center B-cell-like; n, number of patients. Either Pearson’s χ^2^ or Fisher’s exact tests were used

Gene alterations between the CNS relapse (Cohort B) and non-relapse (Cohort C) groups were compared, and are listed in Table 3. *MYD88 L265P (p* = 0.006*), ARID1A mutation (p* = 0.032) and *ETS1* mutation (*p* = 0.015) were more commonly observed in Cohort B than in Cohort C (Table 3). Copy number variation (CNV) in *CDKN2A* (19.5 vs. 3.0%, *p* < 0.001), *BTG1* (9.8 vs. 0%, *p* = 0.001), *IRF8* (7.3 vs. 0%, *p* = 0.007), and *UBR5* (7.3 vs. 0.6%, *p* = 0.024) occurred more frequently in cohort B than in cohort C (Table 3). Notably, a correlation between *MYD88* L265P and *CDKN2A* was observed in patients without CNS involvement at the time of diagnosis. The co-occurrence of *MYD88* L265P and *CDKN2A* was significant in both Cohorts B (odds ratio [OR]: 12.0, *p* = 0.007) and C (OR: 11.48, *p* = 0.034). However, this correlation was not observed in Cohort A (OR: 7.00, *p* = 0.130).

**Table 3:** Gene alterations in patients with DLBCL, stratified by CNS involvement

Gene	CNS involvement stratification			*p*-value	
	Cohort A (*n* = 24) No. (%)	Cohort B (*n* = 41) No. (%)	Cohort C (*n* = 169)No. (%)	Cohort A vs Cohort B	Cohort C vs Cohort B
*MYD88*	10 (41.7)	11 (26.8)	26 (15.4)	0.275	0.108
*MYD88 *L265P	9 (37.5)	9 (22.0)	11 (6.5)	0.251	0.006
*MLL2*	5 (20.8)	11 (26.8)	41 (24.3)	0.767	0.840
*PIM1*	5 (20.8)	10 (24.4)	30 (17.8)	1.000	0.376
*CD79B*	4 (16.7)	5 (12.2)	7 (4.1)	0.715	0.061
*ARID1A*	2 (8.3)	8 (19.5)	12 (7.1)	0.301	0.032
*HIST1H1E*	3 (12.5)	3 (7.3)	18 (10.7)	0.662	0.772
*CARD11*	2 (8.3)	5 (12.2)	12 (7.1)	1.000	0.335
*BTG1*	2 (8.3)	2 (4.9)	7 (4.1)	0.622	0.689
*PHF6*	1 (4.2)	3 (7.3)	4 (2.4)	1.000	0.137
*LIN54*	1 (4.2)	2 (4.9)	2 (1.2)	1.000	0.172
*ZFAT*	0	2 (4.9)	2 (1.2)	0.527	0.172
*IKBKB*	0	3 (7.3)	3 (1.8)	0.290	0.090
*FUBP1*	0	3 (7.3)	5 (3.0)	0.290	0.189
*BTBD3*	0	3 (7.3)	2 (1.2)	0.290	0.052
*TET2*	0	5 (12.2)	7 (4.1)	0.149	0.061
*ETS1*	0	5 (12.2)	4 (2.4)	0.149	0.015
*DDX10*	0	0	8 (4.7)	1.000	0.360
*INO80*	0	5 (12.2)	7 (4.1)	0.149	0.061
*CDKN2A *loss	4 (16.7)	8 (19.5)	5 (3.0)	1.000	< 0.001
*BTG1 *gain	0	4 (9.8)	0	0.288	0.001
*IRF8 *gain	0	3 (7.3)	0	0.290	0.007
*UBR5 *gain	0	3 (7.3)	1 (0.6)	0.290	0.024
*ZFAT *gain	0	3 (7.3)	3 (1.8)	0.290	0.090

n, number of patients. Either Pearson’s χ^2^ test or Fisher’s exact tests were used

### Risk factors for secondary CNS occurrence

To investigate the risk factors of CNS relapse, univariate analysis was done including data from those patients with an incidence rate ≥ 5% and the clinical factors from Cohorts B + C. We found that the IPI groups, Ann Arbor stage, ECOG PS, LDH, multiple extranodal involvement, B symptoms at diagnosis, response to initial therapy, *CDKN2A* loss, *MYD88* L265P, co-occurrence of *MYD88 *L265P and *CDKN2A* loss, and mutations in *INO80*, *ARID1A*, and *TET2* correlated with higher frequencies of CNS relapse (Table 4). Besides, the COO classification using RNA-seq tended to be related to CNS relapse risk without statistical significance (*p* = 0.131). IPI groups, COO, B symptoms at diagnosis, and the above gene alterations were used in a multivariate analysis. This multivariate logistic regression analysis revealed that concomitant *MYD88 *L265P and *CDKN2A* loss (*p* = 0.012), *TET2* mutation (*p* = 0.037), *ARID1A* mutation (*p* = 0.010), and *INO80* mutation (*p* = 0.002) were independently correlated with high risk of CNS relapse, after adjusting for the IPI risk groups, B symptom and COO (Table 5).

**Table 4:** Univariate analysis of risk factors for CNS relapse in patients with DLBCL

Factor	OR	95% CI	*p*-value
IPI groups	0.020	0.003–0.164	< 0.001
Ann Arbor stage	10.210	3.004–34.699	< 0.001
ECOG PS	4.434	2.050–9.590	< 0.001
LDH	4.337	1.705–11.031	0.002
Multiple extranodal involvement	3.692	1.630–8.362	0.002
B symptoms at diagnosis	6.569	2.968–14.540	< 0.001
Response to initial therapy	5.960	1.138–31.203	0.035
*CDKN2A* loss	8.452	2.501–28.564	0.001
*MYD88* L265P	3.700	1.319–10.384	0.013
Co-occurrence of *MYD88* L265P and *CDKN2A* loss	11.067	1.940–63.137	0.007
*INO80*	3.966	1.178–13.349	0.026
*ARID1A*	4.000	1.492–10.725	0.006
*TET2*	3.966	1.178–13.349	0.026
*ZFAT *gain	5.323	1.027–27.595	0.046

*CI* confidence interval, *ECOG PS* Eastern Cooperative Oncology Group performance status, *IPI* international prognostic index, *LDH* lactate dehydrogenase, *OR* odds ratio

**Table 5:** Multivariate analysis of risk factors for CNS relapse in patients with DLBCL

Factor	OR	95% CI	*p*-value
IPI groups			
High	1	-	-
Medium	0.699	0.171–2.850	0.617
Low	0.029	0.002–0.427	0.010
COO (RNA-seq)			
ABC	1	-	-
GCB	0.459	0.112–1.885	0.280
Unclassified	0.299	0.041–2.187	0.234
B symptoms at diagnosis	4.810	1.280–18.073	0.020
*ARID1A*	10.532	1.775–62.496	0.010
Co-occurrence of MYD88 L265P and CDKN2A	36.526	2.201–606.236	0.012
*TET2*	18.854	1.191–298.424	0.037
*INO80*	24.789	3.195–192.344	0.002

A logistic regression analysis was performed

*ABC* activated B cell-like, *CI* confidence interval, *COO* cell of origin, *GCB* germinal center B cell-like, *IPI* international prognostic index, *OR* odds ratio

**Fig. 2:**
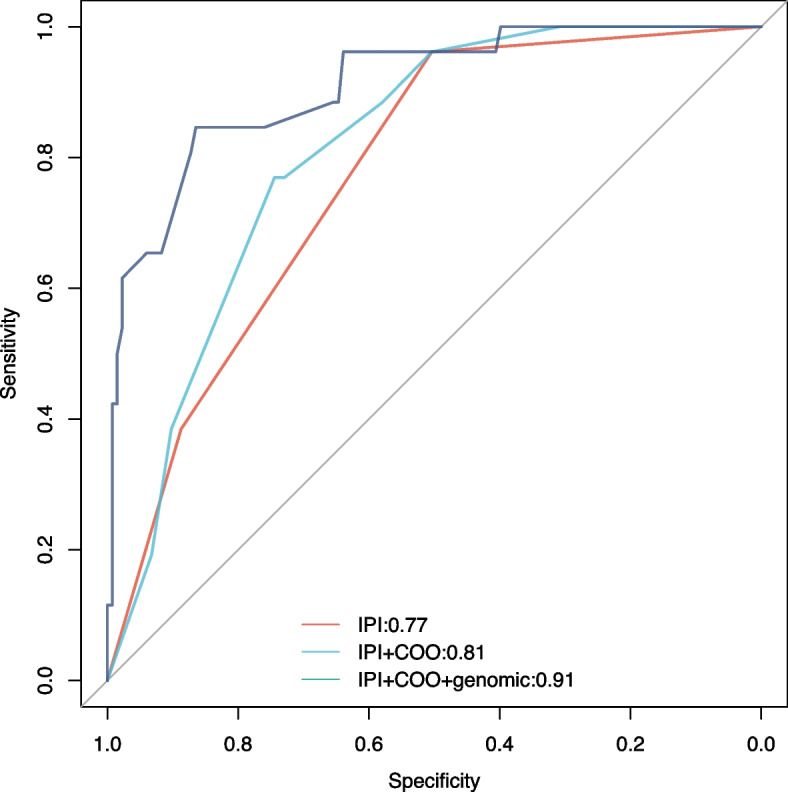
A receiver operating characteristic curve for CNS relapse in patients with DLBCL was constructed using a classifier that integrated genomic risk factors, International Prognostic Index (IPI), and cell of origin (COO) data

We further included the five genes listed in the multivariate model in a genomic classifier. The classifier that integrated genomic risk factors was able to predict CNS relapse with a higher AUROC (area under the receiver operating characteristic curve: 0.91) than those calculated using the IPI (AUROC: 0.77, *p* < 0.001) or the IPI in combination with COO classifiers (AUROC: 0.81, *p* = 0.013) (Fig. 2), and was statistically significant.

## Discussion

The CNS-IPI was developed and validated using several post-rituximab datasets and is recommended for predicting CNS relapse in DLBCL [22]. However, it is somewhat disappointing to have an approximately 10% risk of CNS relapse in high-risk groups, which is highly underestimated. One possible way to optimize the risk estimation for CNS relapse relies on the integration of biomarkers. Apparently, prophylactic strategies for treating patients with DLBCL, such as intrathecal chemotherapy or high-dose methotrexate, have shown some benefits, but these results are somewhat controversial due to the low CNS recurrence rate and the retrospective nature of the studies [12, 14, 18, 19]. Therefore, improvements in risk stratification and patient selection could facilitate clinical trial design and might result in the precise prescription of active and safe CNS prophylactic treatments in high-risk patients. In the current study, we suggest that mutations in *TET2*, *ARID1A*, and* INO80* as well as concomitant *CDKN2A* loss and *MYD88* L265P are independently correlated with high risk of CNS recurrence.

Recently, 1418 patients with DLBCL, from a phase III GOYA study, were analyzed for CNS relapse [28]. COO assessed by GEP, dual expression assessed by BCL2 and MYC immunohistochemistry, and CNS-IPI were evaluated using a multivariate Cox regression model, which demonstrated that high CNS-IPI and ABC/unclassified COO were associated with a higher CNS relapse risk [28]. Based on this advancement, a modified risk stratification model, termed CNS-IPI-C, was created, with a predicted 15.2% risk of CNS relapse at 2 years in the high-risk group [28]. Our study investigated the predictive value of the COO classification for secondary CNS events. Despite the significant correlation between COO and CNS relapse in the univariate analysis, we failed to confirm an independent association between COO classification and CNS relapse after integrating genomic alterations. This could be related to the distribution of mutations among the COO subgroups.

A previous study suggested an unbalanced distribution of *ARID1A* in different COO subtypes [30]. Unfortunately, the GEP-based COO classification is usually not applicable or affordable in routine clinical practice. In our study, the classifier that integrated genomic risk factors was superior in predicting CNS relapse compared with those classifiers including the IPI or the IPI in combination with COO. Therefore, applying next-generation sequencing (NGS) to baseline tumor tissues to identify high-risk of CNS recurrence in patients with DLBCL is of paramount research interest. Nevertheless, limited by available clinical and genomic information, prospective studies are warranted to validate these results.

Consistent with previously published data, *MYD88* mutations were most frequently observed in PCNSL and secondary CNS lymphomas [31]. Previous whole-exome sequencing and phylogenetic analyses of PCNSL have also suggested that *MYD88* L265P mutation and *CDKN2A* loss are early clonal events in PCNSL evolution [29]. Although no significant difference was observed between *MYD88* L265P and *CDKN2A* loss in patients with CNS involvement at diagnosis, the correlation was significant in patients without initial CNS involvement. According to Klanova et al. and Suzuki et al., *CDKN2A* and *MYD88* mutations are more common in CNS-relapsed population, but no significant correlation was determined in multivariate analysis. Notably, the missing NGS data in these studies limited further analysis [28, 33]. In our study, both *CDKN2A* loss and *MYD88 *L265P were more frequently observed in patients with CNS relapse than in those without relapse. We further include concomitant *CDKN2A* loss and *MYD88 *L265P in the multivariate analysis to predict secondary CNS events and found a significant relationship. These results suggest the possibility of investigating the activity of CDK4/6 inhibitors in patients with DLBCL and CNS events.

Our study has some limitations. First, information about kidney/adrenal gland involvement is not available, which hinders the comparison between the CNS-IPI and the newly developed model. Second, the results of this study may have been confounded by missing data such as the time from diagnosis/treatment to CNS events. Third, the current study may be biased because of the lack of CNS prophylaxis information for the patients. Finally, owing to the retrospective nature of this study and the absence of validation, the results warrant validation and prospective exploration.

## Conclusions

The current study revealed a variety of genomic risk factors for predicting CNS relapse in patients with DLBCL. With the development of novel targeted drugs and advances in the biological behavior of DLBCL, individually tailored prophylactic treatments are expected to mitigate secondary CNS events.

## Supplementary Information

Supplementary Material 1.

Supplementary Material 2.

## Data Availability

No datasets were generated or analysed during the current study.

## Abbreviations

ABC: Activated B cell-like
CHOP: Cyclophosphamide, doxorubicin, vincristine, and prednisone
CNS: Central nervous system
CNS-IPI: Central nervous system International Prognostic Index
CNV: Copy number variation
COO: Cell of origin
DLBCL: Diffuse large B-cell lymphoma
ECOG: Eastern Cooperative Oncology Group
FISH: Fluorescence in situ hybridization
FPKM: Fragments per kilobase
GCB: Germinal center B cell-like
GEP: Gene expression profiling
IPI: International Prognostic Index
LDH: Lactate dehydrogenase
NCCN: National Comprehensive Cancer Network
OS: Overall survival
PCNSL: Primary central nervous system lymphoma
PS: Performance status
SNV: Single nucleotide variation
